# Identification of a Ten-Gene Signature of DNA Damage Response Pathways with Prognostic Value in Esophageal Squamous Cell Carcinoma

**DOI:** 10.1155/2021/3726058

**Published:** 2021-12-22

**Authors:** Weitao Zhuang, Xiaosong Ben, Zihao Zhou, Yu Ding, Yong Tang, Shujie Huang, Cheng Deng, Yuchen Liao, Qiaoxia Zhou, Jing Zhao, Guoqiang Wang, Yu Xu, Xiaofang Wen, Yuzi Zhang, Shangli Cai, Rixin Chen, Guibin Qiao

**Affiliations:** ^1^Department of Thoracic Surgery, Guangdong Provincial People's Hospital, Guangdong Academy of Medical Sciences, Guangzhou 510080, China; ^2^Shantou University Medical College, Shantou 515041, China; ^3^The Second School of Clinical Medicine, Southern Medical University, Guangzhou 510515, China; ^4^Burning Rock Biotech, Guangzhou 510300, China; ^5^Research Center of Medical Sciences, Guangdong Provincial People's Hospital, Guangdong Academy of Medical Sciences, Guangzhou 510080, China

## Abstract

Molecular prognostic signatures are critical for treatment decision-making in esophageal squamous cell cancer (ESCC), but the robustness of these signatures is limited. The aberrant DNA damage response (DDR) pathway may lead to the accumulation of mutations and thus accelerate tumor progression in ESCC. Given this, we applied the LASSO Cox regression to the transcriptomic data of DDR genes, and a prognostic DDR-related gene expression signature (DRGS) consisting of ten genes was constructed, including *PARP3, POLB, XRCC5, MLH1, DMC1, GTF2H3, PER1, SMC5, TCEA1,* and *HERC2*. The DRGS was independently associated with overall survival in both training and validation cohorts. The DRGS achieved higher accuracy than six previously reported multigene signatures for the prediction of prognosis in comparable cohorts. Furtherly, a nomogram incorporating DRGS and clinicopathological features showed improved predicting performance. Taken together, the DRGS was identified as a novel, robust, and effective prognostic indicator, which may refine the scheme of risk stratification and management in ESCC patients.

## 1. Introduction

Esophageal cancer, with 604,100 new cases accounting for 544,076 deaths in 2020, ranked fifth among the most common deadly gastrointestinal carcinomas [1]. Based on the histopathological manifestations, esophageal cancer is classified into two main subtypes, esophageal adenocarcinoma (EAC) and esophageal squamous cell carcinoma (ESCC). EAC has a higher prevalence in Western countries, while ESCC is more common in the developing world, including Asia [2]. This geographical bias has been suspected to be related to environmental differences and genetic factors. Irrespective of the heterogeneity of patients with esophageal cancer, the five-year relative survival rate for all stages combined remains less than 20%, thus putting this particular cancer into the group with the worst prognostic outcomes [3].

Although persistent efforts have been made to determine the prognostic factors of esophageal cancer, however, the prognostic analyses based on the clinical characteristics and assay of traditional serum biomarkers, such as squamous cell carcinoma antigen (SCC Ag) [4], cytokeratin 19 fragments CYFRA 21-1 [5], and vascular endothelial growth factors [6], exhibited limited predictive power. In recent years, the development of the next-generation genome sequencing technologies and open-source cancer gene expression profiles, such as The *Cancer* Genome Atlas (TCGA) project, has dramatically advanced the field of data mining-based studies to identify novel prognostic biomarkers. Thus, recent studies have profoundly explored the wide ranges from single gene marker to multigene array for the potential mRNA [7, 8], long noncoding RNA (lncRNA) [9], and competing endogenous RNA (ceRNA) network [10]-based prognostic biomarkers for esophageal cancer. In addition to the genes, including *FAM46A*, *RAB15*, *SLC20A1*, *IL1A*, and *ACSL1*, which have been found to be associated with the overall survival (OS) or relapse-free survival (RFS) of EAC patients [11], several autophagy-related genes [12], as well as glycolysis-related genes [13], have also been detected as the potential prognostic biomarkers of EAC progression. Moreover, ceRNA network-derived eight-gene panel [10] and four-gene panel [13] models have been established to predict the overall survival rate of EAC patients. Furthermore, several genetic panels have been developed based on the tumor microenvironment-associated oncogenes [14, 15], flavoproteins [16], histone modifications [17], actin cytoskeletal proteins [18], and other heterogeneous pathways [19–21], to track the ESCC prognostic signatures. However, a substantial fraction of these models, especially those for ESCC, exhibited unsatisfactory, moderate, or uneven prediction performance without lateral comparisons, which further warrants developing novel biomarkers with higher precision.

Among the various esophageal cancer-related genes, those involved in the DNA damage response (DDR) pathway have shown potential prognostic values. The DDR signaling is critical for initiating DNA damage repair processes to maintain the genomic integrity, which, if otherwise compromised, may lead to the accumulation of aberrant genetic changes and can transform normal cells into cancer cells by activating oncogenes [22]. Earlier studies have developed DDR-related gene panels for the prognostic analysis in different cancers, including ovarian cancer [23], glioblastoma [24], and low-grade gliomas [25]. However, the number and function of genes vary widely across different models, indicating the involvement of a broad spectrum of DDR-related genes in the prognosis of various tumors. Given that ESCC is primarily associated with DDR dysfunction and carcinogen-specific genetic mutations, we hypothesized that there might be a potential correlation between modulation of the DDR-related gene expression and the prognosis in ESCC patients. This correlation could be regarded as the basis for predicting novel signatures for ESCC prognosis, which might exhibit better performance than the existing models.

In this study, we aimed to build a prognostic signature based on the mRNA expression of DDR genes for ESCC. A pre-optimized 10-gene panel predictive model for ESCC prognostic signature was established and validated in an integrated cohort from the Gene Expression Omnibus (GEO) and TCGA databases.

## 2. Materials and Methods

### 2.1. Data Curation

mRNA expression profiles of tumor tissue samples from 2 publicly available ESCC cohorts, including a gene microarray data set (GSE53625 cohort) from the GEO database and an RNA sequencing data set from the TCGA-ESCC cohort, were retrieved. Only patients with pathologically confirmed ESCC diagnoses were included in this study [26].

For microarray data from the GEO database, the normalized matrix file was directly downloaded. RNA sequencing data file (count values) of TCGA was downloaded from the Genomic Data Commons (GDC, https://portal.gdc.cancer.gov/) using the *R* package GDCRNATools [27]. The trimmed mean of M value (TMM) algorithm was used to normalize the count values, and then, the resultant values were transformed to log2 counts per million (logCPM) of transcripts [28]. The batch effect caused by nonbiological technical biases was reduced using the “ComBat” algorithm [29]. All data sets were column-combined and then randomly split into three balanced subgroups: meta-training, meta-testing, and meta-validation data sets.

Transcriptomic data sets and clinical information for 28 cancers in TCGA and three immunotherapy cohorts, including metastatic urothelial carcinoma (IMvigor210) [30] treated with atezolizumab and metastatic melanoma (Liu2019 and GSE78220) [31, 32] treated with pembrolizumab and nivolumab, were analyzed to determine the immunotherapy prognostic value of the DDR-related gene expression signature (DRGS) model.

### 2.2. Generation of DDR-Related Gene Expression Signature (DRGS) Model

A prognostic signature model based on the previously reported 276 DDR genes was constructed [33]. Briefly, a prognostic signature was developed using the meta-training set. To minimize the risk of overfitting, integration of best subset regression into LASSO Cox analyses was applied to identify a panel of genes followed by the construction of the multigene prognostic signature for predicting OS in the meta-training set. Subsequently, based on the above prognostic genes, a formula was defined to calculate the DRG score for each patient as follows: sum (gene's coefficient × each gene's expression level).

### 2.3. Validation of the Prognostic Signature

To validate the classification effect of the signature, we applied the DRG score in the meta-training set, meta-testing set, and meta-validation set, respectively. Patients in these three sets were grouped into the low- and high-risk groups using the cutoff value obtained from the meta-training set. The performance of the DRG score was evaluated by the Kaplan–Meier (KM) survival analysis, the area under the curve (AUC) of the receiver operating characteristic (ROC), and the C-index.

The independent prognostic value of the DRG score was assessed in the GSE53625 and TCGA-ESCC cohorts using univariate and multivariate analyses, respectively. All available clinicopathological variables, such as age, sex, smoking habit, tumor grade, cancer stage, tumor location, and frequency of alcohol consumption, were included.

We further built a nomogram integrating the independent prognostic factors, including the DRG score and clinical factors in the multivariate analyses, for prognostic prediction for patients with ESCC. The prediction efficiency of the nomogram was evaluated by C-index and calibration curves. Decision curve analysis (DCA) was employed to determine the clinical value of the nomogram.

Finally, the predictive accuracy of the DRG score was compared with previously published multigene signatures in both C-index and AUC.

### 2.4. Gene Set Enrichment Analyses (GSEAs)

To evaluate the function of DRG score, GSEA was performed on Molecular Signatures Database v.7.2 using GSEA v.4.1.0. to identify pathways that were enriched in the high- or low-risk group.

### 2.5. Statistical Analysis

Wilcoxon's rank-sum test and chi-square test were applied for continuous variables and categorical variables, respectively. The createDataPartition function in the caret package was used to create balanced splits of target data. The LASSO Cox regression and the best subset regression were performed by the glmnet package and leaps package, respectively [34, 35]. Genes were selected using the method described by Zhou et al. [36]. The log-rank test was applied to compare the survival curves of two or more groups. Univariable and multivariable analyses were performed using the Cox proportional hazards model. The restricted mean survival (RMS) curves were compared for the survival distribution using survRM2 R packages. All data preprocessing, statistical analyses, and graphics were performed in *R* software v4.0.2. *P* < 0.05 was regarded as statistically significant.

## 3. Results

### 3.1. Generation of DDR-Related Gene Signature

A total of 259 primary ESCC tumors were divided into three groups: a meta-training set (*n* = 104), a meta-testing set (*n* = 78), and a meta-validation data set (*n* = 77). The baseline characteristics are summarized in Table S1. The LASSO Cox analysis was performed in the meta-training set and selected ten DDR genes when the best lambda of 0.072 was chosen. As shown in Figure S1, a DRGS consisting of 10 genes *(PARP3, POLB, XRCC5, MLH1, DMC1, GTF2H3, PER1, SMC5, TCEA1,* and *HERC2)* from six pathways was designed using the best subset regression analysis. The DRG scores were calculated as follows:

(1) $$\begin{array}{c} \text{DRGscore}=\left(0.4682*\text{Exp}\left(\text{PARP}3\right)\right)-\left(0.1762*\text{Exp}\left(\text{POLB}\right)\right)\\ +\left(1.8435*\text{Exp}\left(\text{XRCC}5\right)\right)-\left(0.3486*\text{Exp}\left(\text{MLH}1\right)\right)\\ -\left(0.4595*\text{Exp}\left(\text{DMC}1\right)\right)-\left(0.8351*\text{Exp}\left(\text{GTF}2\text{H}3\right)\right)\\ -\left(0.4864*\text{Exp}\left(\text{PER}1\right)\right)+\left(0.1528*\text{Exp}\left(\text{SMC}5\right)\right)\\ -\left(0.9118*\text{Exp}\left(\text{TCEA}1\right)\right)-\left(0.1677*\text{Exp}\left(\text{HERC}2\right)\right). \end{array}$$

A score of 1.32 was used as a cutoff value based on the DRG score of the meta-training set and was applied to all subsequent stratifications.

### 3.2. Association between DRGS and Survival across Different Data sets

**Figure 1:**
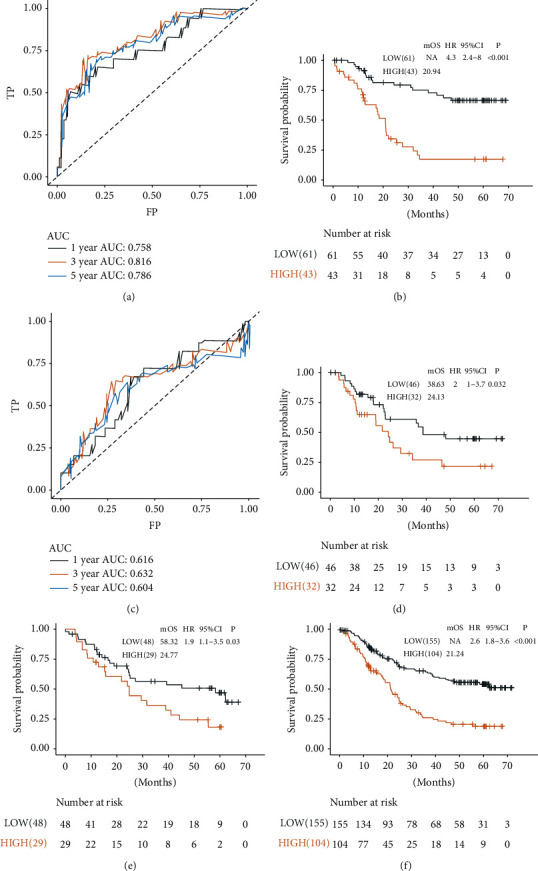
Performance of the DRGS in predicting OS in the training, testing, and validation sets. (a) Time-dependent ROC analyses at 1-, 3-, and 5-year survival rates of the DRGS in the meta-training set.(b), (c) KM analysis of the DRGS in the meta-training set. (d) Time-dependent ROC analyses at 1-, 3-, and 5-year survival rates of the DRGS in the meta-testing set. (d) KM analysis of the DRGS in the meta-testing set. KM analysis of the DRGS in (e) the meta-validation set and (f) the whole meta-data sets.

First, the prognostic prediction performance of the DRGS was estimated in the meta-training and meta-testing sets. In the meta-training set, there were 43 patients in the high-risk group. The AUCs for the 1-, 3-, and 5-year OS of the meta-training set were 0.758, 0.816, and 0.786, respectively (Figure 1(a)). Patients in the high-risk group exhibited shorter OS than the ones in the low-risk group (*P* < 0.001; HR = 4.3; 95% confidence interval (CI) = 2.4–8.0, Figure 1(b)). Similar results were also observed in the meta-testing set, where the AUCs for 1-, 3-, and 5-year OS were 0.614, 0.632, and 0.604, respectively (Figure 1(c)). The OS was also shorter in the patients with higher risk than those with lower risk (*P*=0.032; HR = 2.0; 95% CI = 1.0–3.7, Figure 1(d)).

To further confirm the prognostic value of the DRGS, we validated the DRGS score in other data sets. Using the preestablished cutoff of the risk score in the meta-training sets, 29 patients were identified as high risk in the meta-validation sets (*P*=0.03; HR = 1.9; 95% CI = 1.1–3.5, Figure 1(e)). The AUCs for 1-, 3-, and 5-year OS in the meta-validation sets were 0.536, 0.588, and 0.626, respectively. Considering the sample size, we further explored the association between DRGS and survival in the merged meta-cohort, and similar results were obtained (Figure 1(f)). Similar analyses were conducted in both GSE53625 and TCGA-ESCC cohorts, and similar results were achieved (Figure S2).

### 3.3. Independence, Subgroup, and Comparative Analysis

We then verified the independent prognostic performance of the DRGS using multivariable Cox regression analysis by adjusting clinicopathological factors as previously mentioned. In the multivariable analysis, including the variables with significant results in the univariable analysis (*P* < 0.05), DRGS was identified as an independent prognostic factor for OS (*P* < 0.001; HR = 2.67; 95% CI = 1.80–3.94, Table 1). The American Joint *Cancer* Committee (AJCC) guided the tumor, node, metastasis (TNM) staging system, and tumor location and grade are regarded as the standard diagnostic factors for predicting prognostic outcomes of esophageal cancer. Hence, we included the grade and location values into the multivariable Cox regression analysis, which showed a significant association between DRGS and OS (*P* < 0.001; HR = 2.52; 95% CI = 1.69–3.75, Table 1).

**Figure 2:**
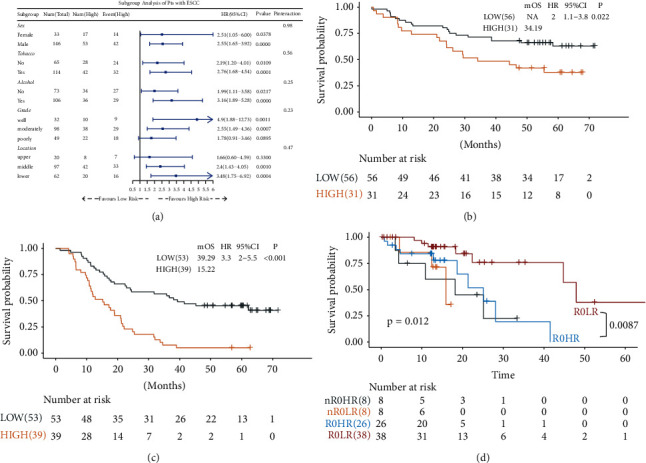
Performance of the DRGS in predicting OS among clinical factors. (a) Subgroup analyses estimating the prognostic value of DRGS in different clinical factors. (b) KM analysis of the DRGS in the early-stage (I/II) ESCC patients. (c) KM analysis of the DRGS in the advanced-stage (III/IV) ESCC patients. (d) KM survival curves of OS among four patient groups stratified by the DRGS and residual tumor.

We further performed subgroup analysis to investigate potential confounding factors (sex, tobacco, alcohol, grade, and location). Compared with the low-risk group, high-risk group patients had lower OS rates irrespective of their sex, smoking habits, and drinking habits. In addition, patients in well- and moderate-differentiated status and with middle-lower thoracic ESCC also exhibited shorter OS (Figure 2(a)). According to the TNM staging system for ESCC, patients were separated into two subgroups: early stage (TNM stages I and II) and late stage (TNM stages III and IV). We found equivalent predictive efficacy in patients in both early and late stages (Figures 2(b)–2(c)). We then specifically examined the ability of the DRGS combined with the residual tumor in patients with ESCC. We found that patients with low DRGS and R0 resection margin status had significant survival advantages, while patients with high DRGS, even with R0 resection margin status, had a worse OS compared with those without R0 resection margin status (Figure 2(d)).

**Figure 3:**
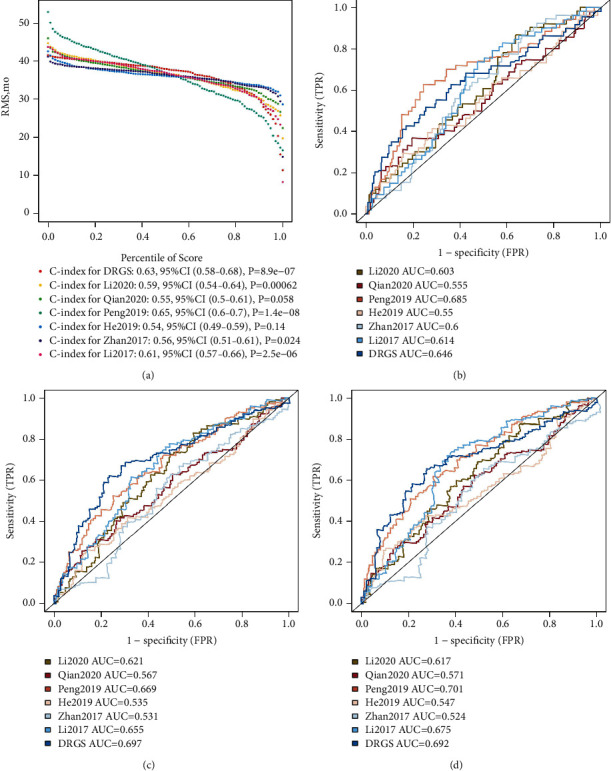
Performance comparison between the DRGS and six previous models. Comparison of the DRGS with previously published signatures using restricted mean survival (RMS) time (a) and AUC for predicting 1-year (b), 3-year (c), and 5-year (d) survival rates.

Furthermore, we compared the DRGS with several previously published multigene signatures for predicting ESCC prognosis using AUC and C-index. The results demonstrate that the DRGS showed a comparable C-index and AUC for OS prediction than the other six signatures (Figure 3, Table S2).

### 3.4. Association between DRGS and Cancer Hallmarks

**Figure 4:**
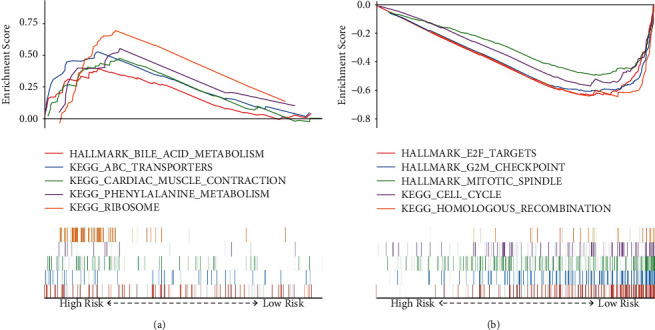
Gene set enrichment analyses between the high- and low-risk groups. Representative hallmarks in (a) the high‐risk group and (b) the low-risk group.

To identify the biological significance of the DRGS, GSEA was conducted to compare the high-risk group with the low-risk group in the TCGA data set. As indicated in Figure 4 and Table S3, cell cycle, G2M checkpoint, E2F targets, mitotic spindle, and homologous recombination pathways were significantly enriched in the low DRGS group, while patients with high DRGS scores showed enrichment of genes involved in the metabolic processes, ribosome synthesis, cardiac muscle contraction, and ABC transporter expression.

### 3.5. Construction and Verification of the Predictive Nomogram

**Figure 5:**
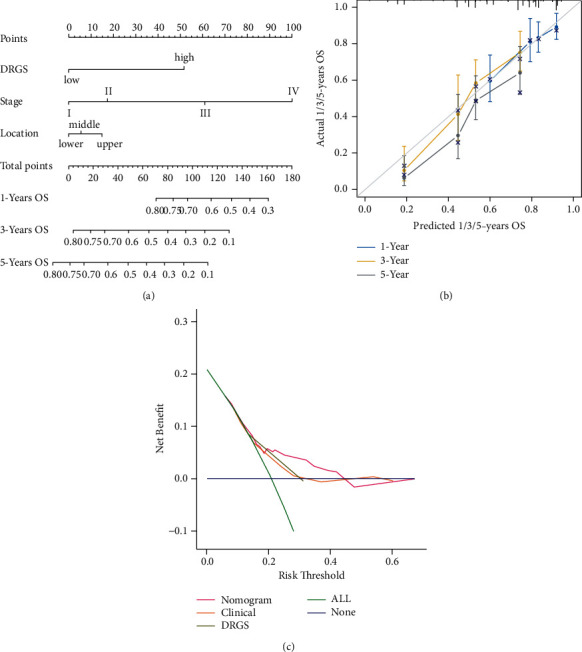
Construction and validation of a nomogram for predicting OS. (a) Nomogram predicting OS for ESCC patients at 1, 3, and 5 years. (b) Calibration plot for predicting 1-, 3-, and 5-year OS. (c) The DCA curves of the nomograms in ESCC.

Next, a nomogram was constructed to predict the 1-, 3-, and 5-year OS rates in ESCC patients integrating DRGS and two clinical factors, including tumor location and tumor stage (Figure 5(a)). Calibration plots indicate that the nomogram might overestimate the 3- or 5-year survival rate (Figure 5(b)). The C-index for tumor stage, tumor location, DRGS, and the nomogram was 0.61 (95% CI = 0.56–0.66), 0.51 (95% CI = 0.47–0.56), 0.61 (95% CI = 0.57–0.65), and 0.67 (95% CI = 0.62–0.72), respectively. The DCA curves demonstrate that the nomogram showed the best net benefit than other factors (Figure 5(c)).

### 3.6. The DRGS in the Prediction of TCGA Pan-Cancer and Immune Checkpoint Inhibition (ICI) Cohorts

**Figure 6:**
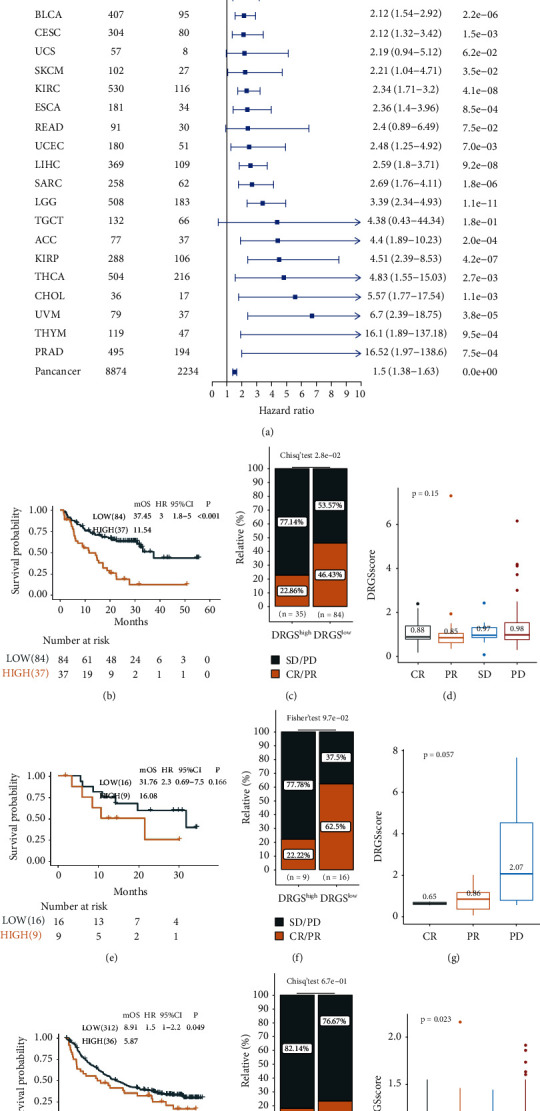
DRGS is a prognostic biomarker and predicts immunotherapy efficacy. Subgroup analyses estimating the prognostic value of DRGS in (a) pan-cancers from TCGA data sets. The Kaplan–Meier survival curves of overall survival in (b) the Liu2019 cohort, (e) the IMvigor210 cohort, and (h) the GSE78220 cohort. Rate of CR/PR and SD/PD to anti-PD-L1 immunotherapy in the high or low group in (c) the Liu2019 cohort, (f) the IMvigor210 cohort, and (i) the GSE78220 cohort. Distribution of DRGS scores with different anti-PD-L1 clinical responses in (d) the Liu2019 cohort, (g) the IMvigor210 cohort, and (j) the GSE78220 cohort. The values represent the mean value. The differences among groups were compared using the Kruskal–Wallis test.

To further examine the utilization of the DRGS, we calculated the DRGS score in the TCGA pan-cancer data set to verify its prognostic value. Although there was heterogeneity among different tumors, the DRGS was supported as a favorable prognostic signature in TCGA pan-cancer (Figure 6(a)).

We next investigated the prognostic value of the DRGS in three immunotherapy cohorts. In both metastatic melanoma (Liu 2019) and metastatic urothelial carcinoma (IMvigor210), the patients with low DRGS scores exhibited favorable clinical benefits and longer survival (Liu 2019, *P* < 0.001, HR = 3.0, 95% CI = 1.80–5.00, Figures 6(b)–6(d); IMvigor210, *P*=0.049, HR = 1.5, 95% CI = 1.00–2.20, Figures 6(h)–6(j)). No significant difference in the GSE78220 cohort was observed (*P*=0.166, HR = 2.3, 95% CI = 0.69–7.50, Figures 6(e)–6(g)), which could be due to the small sample size in this cohort (*n* = 25).

## 4. Discussion

In this study, we developed and validated a DRGS based on the DDR-related genes' expression modulation to predict the prognostic outcomes in patients with ESCC. A nomogram based on the DRGS score and clinical variables was further built for the prognostic prediction. Lastly, our study suggested that the DRGS score was correlated with survival in most tumors beyond ESCC, further proving the potential utility of DRGS in clinical settings. However, the precise clinical application of the DRGS score needs to be further examined in the larger cohorts.

Investigation into the prognostic factors revealed an implication of therapeutic decision guidance, curative efficacy judgment, and prognostic prediction in clinical applications. Conventional prognostic signatures mainly include various clinicopathological risk factors, pathological grade, and TNM stage, which present uneven prediction efficiencies. In this study, the DRGS score displayed robust performance in the prediction of ESCC prognosis, which remained stable in the subgroup analysis and multivariate regression analysis. In addition, it was noteworthy that R0 resection showed significant survival advantages among patients with low DRGS, while R0 resection did not exhibit enhanced survival benefits among patients with high DRGS, which could be indicative of surgical alternatives in clinical application. Considering the poor prognosis in patients with high DRGS, postsurgical adjuvant therapies might be helpful and should be actively considered. The therapeutic benefits of other treatments, such as adjuvant chemotherapy and immunotherapy, needed to be explored in patients with high DRGS. In addition, DRGS is an independent prognostic factor for ESCC, and thus, we constructed an integrated model using DRGS in combination with clinicopathological features, which further improved the predictive performance of the independent factors and supported the clinical utility of DRGS.

Previously published studies have investigated the potential of DDR genes in the prognostic prediction of other cancer types. For example, Pang et al. have developed a DDR-related gene-based prognosis predicting model for low-grade gliomas and subsequently discovered that mutations in the isocitrate dehydrogenase (*IDH*) gene might affect the prognosis through the regulation of DDR pathways [25]. In another study by Sun et al., a prognostic signature was constructed for OS rate prediction in patients with ovarian cancer, which might also serve as a potential therapeutic target in ovarian cancer [23]. Besides, co-mutations in specific DDR pathway-associated genes have been identified as predictors of survival outcomes in response to immune checkpoint blockade, which has inspired the concept of clinical utilization of patient selection for immune therapy [37]. In this study, the 10 DDR-related genes included in the DRGS by LASSO resulted in either positive or negative correlation coefficients, respectively, suggesting that these genes might perform differentially and even with opposite functions in ESCC pathology. As the key interacting partner of RAD51, DNA meiotic recombinase 1 (DMC1) has been reported to promote the proliferation of ESCC cells through the interaction between RAD51 and checkpoint kinase 1 (CHK1) [38]. The downregulation of period circadian regulator 1 (*PER1*) gene expression has been found to enhance tumorigenicity and proliferation of oral squamous cell carcinoma cells [39]. Furthermore, inhibited expression of X-ray repair cross-complementing 5 (*XRCC5*) in ESCC cells has been linked to reduced malignancies of tumor cells, such as proliferation, clonal progression, and apoptosis escape [40]. Besides, our GSEA results also showed significant enrichment of genes involved in cell cycle and mitotic spindle regulations in the low DRGS group and those involved in metabolic process-related pathways in the high DRGS group. Taken together, these findings suggest potential associations between DDR-related genes and regulation of cell division and metabolism, which may affect the occurrence and development of ESCC.

Furthermore, we assessed the application of the DRGS model in pan-cancer cohorts and investigated the potential prognostic value of the DDR-related genes in three immunotherapy cohorts. Results showed that the patients with low DRGS scores exhibited favorable clinical benefits and longer survival after immunotherapy in both metastatic melanoma and metastatic urothelial carcinoma. Possible explanations for such observation could be mutations in DDR-associated genes resulting in a deficiency of their DNA repair capacities, which might, in turn, increase neoantigen burden and subsequently improve the response to immunotherapy. These results could provide novel insights into the promising biomarkers involved in DNA repair pathways for the prediction of responses to immune checkpoint blockade therapies.

However, the limitations of this study should not be ignored. This is a retrospective study based on public databases, which might limit the strength of evidence, and therefore, the results should be regarded as hypothesis-generating rather than conclusive. Besides, DRGS was only validated in one cohort, which might introduce potential bias, and it is recommended to be validated with further cohorts.

## 5. Conclusions

In summary, a DRGS score consisting of 10 DDR-related genes was designed for prognostic prediction in patients with ESCC and validated in two meta-data sets and pan-cancers. Furthermore, a nomogram combing the DRGS score, tumor location, and tumor stage was built, which exhibited great potential in predicting OS and immunotherapy efficacy. Additionally, the DRGS score may conduce to clinical decision-making for treatment and hold promise for clinical practice in the future. Retrospective studies in larger cohorts and prospective studies are warranted to investigate the mechanisms and clinical utility of the DRGS score.

## Figures and Tables

**Table 1:** Univariable and multivariable Cox regression analyses to identify independent prognostic predictors in the GSE53625 cohorts.

Characteristics	Size	Uni-Cox analysis		^a^Multi-Cox analysis		^b^Multi-Cox analysis	
		HR (95% CI)	*P* value	HR (95% CI)	*P* value	HR (95% CI)	*P* value
Age							
≥60 vs. <60	179	1.58 (1.07–2.31)	**0.0240**	1.43 (0.97–2.1)	0.0709	1.57 (1.06–2.34)	0.0235
Sex							
Male vs. female	179	0.78 (0.49–1.25)	0.3070				
Grade							
Moderately vs. well	130	1.01 (0.59–1.75)	0.9620			0.78 (0.44–1.37)	0.3810
Poorly vs. well	81	1.65 (0.93–2.96)	0.0900			1.09 (0.60–1.99)	0.7750
Stage							
II vs. I	179	2.15 (1.45–3.21)	**0.00015**	2.26 (1.51–3.38)	**<0.0001**	2.30 (1.51–3.51)	**0.00011**
Location							
Middle vs. upper	117	0.68 (0.39–1.20)	0.1850			0.64 (0.36–1.16)	0.1400
Lower vs. upper	82	0.60 (0.33–1.11)	0.1010			0.49 (0.26–0.95)	**0.0338**
Tobacco							
Yes vs. no	179	0.75 (0.51–1.10)	0.1450				
Alcohol							
Yes vs. no	179	0.86 (0.59–1.27)	0.4550				
DRGS							
High vs. low	179	2.57 (1.75–3.77)	**<0.0001**	2.67 (1.80–3.94)	**<0.0001**	2.52 (1.69–3.75)	**<0.0001**

a: variables in multi-Cox analysis were selected by *P* < 0.05; b: variables in multi-Cox analysis were selected by *P* < 0.05 and clinical expertise; HR: hazard ratio, CI: confidence interval, DRGS : DDR-related gene expression signature.

## Data Availability

The datasets generated and analyzed during the current study are available in the public data repositories, TCGA-DGC: https://portal.gdc.cancer.gov/repository and GEO: https://www.ncbi.nlm.nih.gov/geo/query/acc.cgi?acc=GSE53625.

## Abbreviations

EAC: Esophageal adenocarcinoma
ESCC: Esophageal squamous cell carcinoma
SCC Ag: Squamous cell carcinoma antigen
TCGA: The *Cancer* Genome Atlas
OS: Overall survival
RFS: Relapse-free survival
DDR: DNA damage response
GEO: Gene Expression Omnibus
GDC: Genomic Data Commons
TMM: Trimmed mean of M values
logCPM: Log2 counts per million
LASSO: Least absolute shrinkage and selection operator
KM: Kaplan–Meier
ROC: Receiver operating characteristic
C-index: Concordance index
DCA: Decision curve analysis
AUC: Area under the curve
GSEA: Gene set enrichment analyses
SE: Standard error
DRGS: DDR-related gene expression signature
AJCC: American Joint Cancer Committee.

## Supplementary Materials

Supplementary Materials
*Supplementary Table S1.* Patient characteristics in the meta-data sets. *Supplementary Table S2*. Comparison of the DRGS with eight other previously reported signatures. *Supplementary Table S3*. GSEA on the TCGA cohort to explore mechanisms underlying the DRGS. *Supplementary Figure S1.* (a) LASSO deviance profiles of the cancer-associated DDR genes in ESCC; (b) LASSO coefficient profiles of the cancer-associated DDR genes in ESCC; (c) the optimal subset selection using best subset regression model; (d) chord diagram for the relationship between signature-based genes and DDR pathways. *Supplementary Figure S2*. Kaplan–Meier analysis of the DRGS in (a) the GSE53625 cohort and (b) the TCGA-ESCC cohort.
