# Supplementary material for: Identification of a Ten-Gene Signature of DNA Damage Response Pathways with Prognostic Value in Esophageal Squamous Cell Carcinoma
Source: J Oncol. 2021 Dec 22;2021:3726058. doi: 10.1155/2021/3726058 (PMC8716225; doi:10.1155/2021/3726058)
Supplement: Supplementary Materials — Supplementary Table S1. Patient characteristics in the meta-data sets. Supplementary Table S2. Comparison of the DRGS with eight other previously reported signatures. Supplementary Table S3. GSEA on the TCGA cohort to explore mechanisms underlying the DRGS. Supplementary Figure S1. (a) LASSO deviance profiles of the cancer-associated DDR genes in ESCC; (b) LASSO coefficient profiles of the cancer-associated DDR genes in ESCC; (c) the optimal subset selection using best subset regression model; (d) chord diagram for the relationship between signature-based genes and DDR pathways. Supplementary Figure S2. Kaplan–Meier analysis of the DRGS in (a) the GSE53625 cohort and (b) the TCGA-ESCC cohort. [file 3726058.f1.zip › 3726058.f1/Supplemental Table S3.doc]

**Table S3** GSEA on the TCGA cohort to explore mechanisms underlying the DRGS.

| **NAME** | **ES** | **NES** | **NOM p-val** | **Group** |
| --- | --- | --- | --- | --- |
| **KEGG_RIBOSOME** | 0.70 | 1.63 | 0.0348 | High risk |
| **KEGG_CARDIAC_MUSCLE_CONTRACTION** | 0.47 | 1.58 | 0.0364 | High risk |
| **KEGG_ABC_TRANSPORTERS** | 0.53 | 1.55 | 0.0500 | High risk |
| **KEGG_ALPHA_LINOLENIC_ACID_METABOLISM** | 0.56 | 1.53 | 0.0405 | High risk |
| **KEGG_OXIDATIVE_PHOSPHORYLATION** | 0.49 | 1.52 | 0.1631 | High risk |
| **KEGG_PARKINSONS_DISEASE** | 0.46 | 1.47 | 0.1605 | High risk |
| **KEGG_PROXIMAL_TUBULE_BICARBONATE_RECLAMATION** | 0.53 | 1.46 | 0.0867 | High risk |
| **KEGG_PHENYLALANINE_METABOLISM** | 0.55 | 1.46 | 0.0405 | High risk |
| **HALLMARK_REACTIVE_OXYGEN_SPECIES_PATHWAY** | 0.51 | 1.45 | 0.1294 | High risk |
| **KEGG_PYRUVATE_METABOLISM** | 0.45 | 1.45 | 0.0751 | High risk |
| **KEGG_PROPANOATE_METABOLISM** | 0.49 | 1.44 | 0.0749 | High risk |
| **HALLMARK_BILE_ACID_METABOLISM** | 0.39 | 1.43 | 0.0448 | High risk |
| **KEGG_HISTIDINE_METABOLISM** | 0.53 | 1.40 | 0.0887 | High risk |
| **KEGG_CELL_CYCLE** | -0.58 | -1.93 | 0.0039 | Low risk |
| **HALLMARK_G2M_CHECKPOINT** | -0.61 | -1.85 | 0.0158 | Low risk |
| **HALLMARK_E2F_TARGETS** | -0.64 | -1.78 | 0.0360 | Low risk |
| **HALLMARK_MITOTIC_SPINDLE** | -0.50 | -1.76 | 0.0405 | Low risk |
| **KEGG_HOMOLOGOUS_RECOMBINATION** | -0.68 | -1.75 | 0.0201 | Low risk |
| **KEGG_MISMATCH_REPAIR** | -0.62 | -1.66 | 0.0508 | Low risk |
| **KEGG_DNA_REPLICATION** | -0.62 | -1.54 | 0.1263 | Low risk |
| **KEGG_OOCYTE_MEIOSIS** | -0.37 | -1.51 | 0.0596 | Low risk |
